# Modification of Collagen Derivatives with Water-Soluble Polymers for the Development of Cross-Linked Hydrogels for Controlled Release

**DOI:** 10.3390/ma12244067

**Published:** 2019-12-06

**Authors:** Ioanna Tzoumani, Georgia Ch. Lainioti, Alexios J. Aletras, Gabriel Zainescu, Simina Stefan, Aurelia Meghea, Joannis K. Kallitsis

**Affiliations:** 1 Department of Chemistry, University of Patras, GR-265 04 Patras, Greece; tzoumani.i@upnet.gr (I.T.); glainioti@upatras.gr (G.C.L.); aletras@chemistry.upatras.gr (A.J.A.); 2 Foundation for Research and Technology-Hellas (FORTH)/Institute of Chemical Engineering Sciences (ICE-HT), Stadiou Str., Platani, P.O. Box 1414, GR-265 04 Rio-Patras, Greece; 3 National R & D Institute for Textile and Leather-Division: Leather and Footwear Research Institute, 93 Ion Minulescu Str., 031215 Bucharest, Romania; gabriel.zainescu@gmail.com; 4 Faculty of Applied Chemistry and Materials Science, Politehnica University of Bucharest, 1-7 Polizu Str., 011061 Bucharest, Romania; simina_stefan_ro@yahoo.com

**Keywords:** collagen hydrolysate, water soluble polymers, cross-linking, epoxide groups, biofertilizers

## Abstract

Novel cross-linked hydrogels were synthesized as potential materials for the development of smart biofertilizers. For this purpose, hydrogels were prepared using collagen hydrolysate recovered from tannery waste. The water-soluble polymer poly(sodium 4-styrenesulfonate-co-glycidyl methacrylate) (P(SSNa-co-GMAx)) was among others used for the cross-linking reaction that combined hydrophilic nature with epoxide groups. The synthetic procedure was thoroughly investigated in order to ensure high percentage of epoxide groups in combination with water-soluble behavior. The copolymer did not show cytotoxicity against normal lung, skin fibroblasts, or nasal polyps fibroblasts. Through the present work, we also present the ability to control the properties of cross-linked hydrogels by altering copolymer’s composition and cross-linking parameters (curing temperature and time). Hydrogels were then studied in terms of water-uptake capacity for a period up to six days. The techniques Proton Nuclear Magnetic Resonance (^1^H NMR), Thermogravimetric Analysis (TGA), Size Exclusion Chromatography (SEC), and Attenuated Total Reflection Fourier Transform Infrared Spectroscopy (ATR-FTIR) were applied for the characterization of the synthesized copolymers and the cross-linked hydrogels. Three samples of biofertilizers based on collagen hydrolysate functionalized with P(SSNa-co-GMAx) and starch and having nutrients encapsulated (N, P, K) were prepared and characterized by physical–chemical analysis and Energy Dispersive X-ray analysis-Scanning Electron Microscope (EDAX-SEM) in terms of microstructure. Preliminary tests for application as fertilizers were performed including the release degree of oxidable organic compounds.

## 1. Introduction

The industries all over the world are the major source of pollution with a significant impact on the environment. Industrial wastes may cause a severe pollution in river systems, lakes, soil, and sea and also atmospheric pollution by fume release. The improper waste management and their disposal to recipients will lead to harmful effects on the biotic and abiotic ecosystems disturbing the ecological balance. Currently, the idea of a circular economy where the waste will be used as a source for useful products is gaining intense interest.

Leather industry has been characterized as one of the most polluting industries due to the high amounts of solid waste and odor pollution coming from leather production and tannery wastewater treatment plants [1]. This will lead to health risks for humans or other living organisms as well as to severe economic and social problems [2]. Thus, the development of innovative and efficient methods to solve the tannery waste problem is still a highly actual issue.

An important part of the tannery waste is rich in type-I collagen, which may be recycled or employed in other industries. The European policies and legislations are encouraging the conversion of tannery waste to collagen derivatives, which can be used in many sectors such as organic fertilizers [3,4], medicine [5], cosmetics [6,7], paper and pulp making [8], biodegradable packaging materials [9], wound healing [10], etc.

Type-I collagen is a water-insoluble protein with a triple-helix structure being of great industrial interest [11,12]. It is characterized by a hydrophilic behavior due to its hydrophilic groups (carboxyl, amino, hydroxyl, and amide groups) [13]. Collagen can be extracted from tanned waste through hydrolysis process. When a partial hydrolysis takes place, gelatin is produced, whereas a stronger hydrolysis results in low molecular weight peptides called collagen hydrolysates.

Collagen hydrolysate in liquid form is difficult to be used for applications like fertilizers mainly due to its odor, the possibility for microbial development, the fast absorption or leaching in the environment as well as the difficulties to be used in the solid form due to the relatively expensive drying process. One option to overcome these problems is the modification of collagen hydrolysate using chemical cross-linking with reactive water-soluble polymeric materials [14,15]. In this approach, a reaction between collagen’s reactive groups and the functional groups of the water-soluble copolymers will lead to the development of chemically cross-linked network hydrogels with enhanced mechanical stability and tunable properties. Additionally, such hydrogels can more easily be isolated, dried and used as solid fertilizers with in situ controllable swelling and release.

Toughening of polymeric hydrogels in combination with high swelling degrees and low release rates has always been a flaming issue for the scientific community [16,17]. It is worth mentioning that novel hydrogel structures have been synthesized by researchers in order to be used in the field of agriculture due to their unique properties, such as the strong hydrophilicity, biocompatibility, and the high swelling capacity. Moreover, the controlled-release systems have been thoroughly investigated by researchers since they improve the efficiency of fertilizers by eliminating the leaching effect. Essawy et al. [18] fabricated superabsorbent polymeric hydrogels through graft polymerization of acrylic acid from chitosan–cellulose hybrid in order to obtain structures with controlled release of nutrients depending on plants’ necessities. Moreover, Li et al. [19] used solution polymerization in order to prepare a novel slow-release fertilizer based on wheat straw semi-interpenetrating polymer networks (semi-IPNs) hydrogel with slow-release nitrogen and phosphorus fertilizers. Calcagnile et al. [20] presented a superabsorbent hydrogel composite material based on biodegradable poly(lactic acid)/cellulose to be used in agricultural and horticultural applications.

It may be noted that protein-based hydrogels, such as collagen derived from industrial by-products, are promising materials for controlled-release applications in agriculture. Copolymers bearing functional groups cross-linked with the collagen matrix will efficiently incorporate the desired nutrients and act as biostimulators in controlled-release fertilizer technology [21,22]. With that knowledge in mind, the development of novel cross-linked hydrogels through the modification of collagen derivatives with water-soluble polymers was an interesting challenge for us. The fact that these materials will create network with high swelling capacity and high gel strength is an important advantage. The release rate of nutrients encapsulated in the polymeric hydrogels could be controlled according to the plants’ needs. Moreover, the non-toxicity of the initial copolymers in combination with the low cost of the final product is an additional motivation for the development of this kind of hydrogels.

In this line, we initially focused on the optimization of the synthesis of the water-soluble copolymer poly(sodium 4-styrenesulfonate-co-glycidyl methacrylate) (P(SSNa-co-GMAx)). The specific copolymer was selected as it combines the behavior of a charged polyelectrolyte and water solubility with the reactive epoxy functional groups, which will further be used for cross-linking reactions. A thorough research regarding the synthetic procedure of the present copolymer was conducted. Moreover, the toxicological impact of the copolymer was assessed. It was tested for cytotoxicity against human normal lung and skin fibroblasts as well as against nasal polyps fibroblasts. In a second part, we focused on the use of polymeric materials for the stabilization of the collagen hydrolysate, by chemical cross-linking either with biopolymers like starch or with polymers bearing epoxide groups. The optimization of the reaction conditions between collagen hydrolysate and P(SSNa-co-GMAx) was conducted in order to obtain hydrogels, which may be encapsulated in organic fertilizers offering the advantages of smart biofertilizers. The formation of the hydrogels was investigated in terms of composition, stoichiometry, curing temperature, and reaction time. By functionalization of collagen hydrolysate with encapsulated nutrients and the synthetic copolymer or starch as biopolymer, preliminary tests for their application as biofertilizers were carried out.

## 2. Materials and Methods

The monomers sodium 4-styrene sulfonate (SSNa), glycidyl methacrylate (GMA), the homopolymers poly(sodium 4-styrenesulfonate) (PSSNa), poly(acrylic acid) (PAA), poly(ethylene oxide) (PEO), copolymers poly(4-styrenesulfonic acid-co-maleic acid), sodium salt P(SSNa-co-MA), the initiator azobisisobutyronitrile (AIBN), as well as deuterium oxide (D_2_O) were purchased from Sigma-Aldrich (Sigma-Aldrich Chemie GmbH, Taufkirchen, Germany) and used as received. The solvents dimethylsulfoxide (DMSO) and acetone were purchased from Fisher Scientific (Fisher Scientific, Pittsburgh, PA, USA) and used as received. Polymer standards poly(ethyleneglycol/ethyleneoxide) (PEO) were purchased from Polymer Laboratories LTD (Polymer Laboratories LTD, Essex Road, Church Stretton, U.K.). Lithium nitrate (LiNO_3_) was purchased from Merck (Merck KGaA, Darmstadt, Germany). The 3-(4,5-dimethylthiazol-2-yl)-2,5-diphenyltetrazolium bromide (MTT) was purchased from Sigma-Aldrich (Sigma-Aldrich Chemie GmbH, Taufkirchen, Germany). The Dulbecco’s modified Eagle’s medium (DMEM) was from BioChrom AG, (BioChrom AG Berlin, Germany). The collagen hydrolysate (7% w/v), used in the present work, was produced according to published procedure [23] and provided by National Research & Development Institute for Textile and Leather (INCDTP, Bucharest, Romania) in the frames of the project AGRO-SMARTGEL (Grant agreement n° 5021383/INCOMERA project). Ultra-pure water was obtained by means of an SG apparatus water purification unit.

### 2.1. Synthesis of P(SSNa-co-GMAx) Copolymers

The copolymers poly(sodium 4-styrene sulfonate-co-glycidyl methacrylate) were synthesized through free radical polymerization using AIBN as initiator according to an experimental procedure previously described [24,25]. In the present study, the solvent used for the reaction was DMSO. The desired quantities of the two monomers (total monomer concentration 1 M) were dissolved in the DMSO, the solution was degassed, and the initiator AIBN (0.8 mol% over the total monomer concentration) was added. The reaction was left to proceed overnight under vigorous stirring in Ar atmosphere in an oil bath set at 80 °C. After cooling down to room temperature, the copolymers were recovered by precipitation in acetone, and filtered and dried in a vacuum oven at 60 °C for 24 h. Copolymers with 2 to 40 mol% GMA content were prepared. The mol fraction of GMA was calculated either from Proton Nuclear Magnetic Resonance (^1^H NMR), using D_2_O as solvent, or through Thermogravimetric Analysis (TGA). The copolymers are denoted as P(SSNa(1 − x)-co-GMAx), where (1 − x) and x are the mol fractions of SSNa and GMA units, respectively, as shown in Table 1, and the results for their molecular characteristics are presented in Table 2.

### 2.2. Modification of Collagen Hydrolysate with P(SSNa-co-GMAx)

The collagen hydrolysate was mixed in different proportions with the copolymer P(SSNa-co-GMAx) at room temperature under stirring conditions. After short time, a viscous mass was precipitated. The precipitated mass was separated from the supernatant solution and remained for different time intervals at room temperature (R.T.) or thermally treated at 80 °C or 120 °C. Finally, both the soluble (water soluble) and insoluble (residue) fractions were dried and then their weights were calculated. The insoluble part was varied between 70 and 100 wt%. The results for selected preparations are shown in Table 3, Table 4, Table 5 and Table 6.

### 2.3. Modification of Collagen Hydrolysate with Encapsulated Nutrients and Polymeric Materials

In this case, the untanned hide waste was treated by hydrolysis of protein waste in acidic medium, resulting in a protein hydrogel, which in combination with other polymers, such as P(SSNa-co-GMAx) and starch, resulted in various hydrogels with a collagen structure.

As an example, 6800 g of pelt waste are weighed, washed, and delimed with 4–4.5% ammonium sulfate, followed by dilution in 5–6.5 L industrial water containing 2–3.5% concentrated H_2_SO_4_, 2.5–3.5% dipotassium phosphate (K_2_HPO_4_∙3H_2_O), and 0.5–1% industrial salt (NaCl). This mixture is poured into a 50 L autoclave with double jacket and shaker, at 80–98 °C for 2.5–4.5 h. This product is gelatinous, elastic, and semi-transparent, with pH of 5.8–6.8, containing collagen hydrogel with encapsulated nutrients (K, P), and will be considered as a reference organic fertilizer for agriculture applications, denoted as CH-Ref [26]. In this stage, 5% copolymer P(SSNa-co-GMA20) with IN20c code is added to obtain a similar fertilizer functionalized with this synthetic copolymer, and will be denoted as CH-IN. If instead of IN20c in the autoclave is added 36–60% corn starch and a methylene bis-acrylamide as chemical bonding agent (0.5 g dissolved in 50 mL water) to form a chemically bounded starch copolymer, a starch graft copolymer is obtained, able to absorb liquid fluids without dissolution. Hydrolysis is continued for 1.5–2 h, at a temperature of 78–85 °C resulting in a superabsorbent hydrogel in the form of a white paste, with a pH about 6.7 and marked as CH-Starch.

### 2.4. Characterization Techniques

#### 2.4.1. Proton Nuclear Magnetic Resonance (^1^H NMR)

The samples for ^1^H NMR characterization were prepared by dissolving the P(SSNa-co-GMAx) copolymers in D_2_O. ^1^H NMR spectra were obtained at 400 MHz at 300 K on a Bruker AVANCE DPX 400 spectrometer (Bruker BioSpin GmbH, Magnet Division, Karlsruhe, Germany). The ^1^H NMR spectra were used to determine the chemical composition of the copolymers.

#### 2.4.2. Thermal Analysis

Thermogravimetric analysis (TGA) was carried out in alumina crucibles in a Labsys™TG (Caluire, France) apparatus of Setaram from 25 to 800 °C under nitrogen and at a heating rate of 10 °C/min.

#### 2.4.3. Size Exclusion Chromatography (SEC)

For the water-soluble precursors P(SSNa-co-GMAx), a Millipore Waters 501 HPLC chromatographer (Milford, MA, USA) at 25 °C was used, equipped with two Shodex B-804, B-805 linear columns (8 mm × 500 mm), a differential refractometer (R401) detector (Milford, MA, USA), poly(ethylene oxide) standards, and 0.1 M LiNO_3_ as eluent. The operating flow rate was set at 1 mL/min.

#### 2.4.4. Attenuated Total Reflection Fourier Transform Infrared Spectroscopy (ATR-FTIR)

The Attenuated Total Reflection Fourier Transform Infrared Spectroscopy (ATR-FTIR) spectra of the copolymers, the collagen hydrolysate, and the collagen-P(SSNa-co-GMA) cross-linked hydrogels were recorded using Bruker Optics’ Alpha-P Diamond ATR Spectrometer of Bruker Optics GmbH (Ettlingen, Germany).

#### 2.4.5. Energy Dispersive X-ray analysis-Scanning Electron Microscope (EDAX-SEM)

Scanning electron microscopy of cross-linked hydrogels (SEM, JEOL 6300, Tokyo, Japan, instrument equipped with an X-ray Energy Dispersive Spectrometer, EDS) was performed to investigate their morphologies. The samples were obtained through freeze-drying and sputtered with gold to produce electric conductivity before SEM examination.

The morphology of collagen hydrolysate with encapsulated nutrients and polymeric materials was conducted through Scanning Electron Microscopy on SEM, Hitachi, S-3400 N type II Model, Secondary Electrons, High Vacuum, 10–3 Pa, 3 nm at 30 kV. The Energy Dispersive X-ray Spectroscopy (EDS/EDX) analysis was performed using Thermo Ultra Dry, Noran System 7, NSS Model (2,000,000 counts/s).

### 2.5. Toxicological Assessment of the Copolymer P(SSNa-co-GMA20)

#### 2.5.1. Cell Lines and Cell Culture Conditions

Lung and skin fibroblasts were isolated from lung and skin tissues, respectively, of healthy donors. Nasal polyps fibroblasts were isolated from polyps’ tissues obtained from patients undergoing sinus surgery for treatment of polyposis. The isolation and culture of fibroblasts were performed as previously described for eye pterygium fibroblasts [27]. All cells were cultured as monolayers in Dulbecco’s modified Eagle’s medium (DMEM), supplemented with 10% heat-inactivated fetal calf serum (FCS), 1% penicillin and streptomycin, in a humidified 5% CO_2_ atmosphere.

#### 2.5.2. Cell Viability

i  Toxicity Test of the Copolymer P(SSNa-co-GMA20)

For cell viability, the tetrazolium/formazan assay was used, as previously described [28]. Fibroblasts in DMEM–10% FCS were seeded onto 96 well plates. When the cell monolayers reached confluent, the conditioned medium was replaced with solutions of copolymer at different concentrations (0.125–100 µg/mL) in the same medium, and the cells were cultured for different time intervals. Then, the conditioned medium was removed and a solution of MTT (1 mg/mL) in the same medium, was added. After incubation at 37 °C for 4 h in a humidified 5% CO_2_ atmosphere, the solution was thoroughly aspirated, the crystals of formazan were dissolved in DMSO, and the absorbance at 540 nm was measured, using a microplate reader (TECAN infinite M200, Grödig, Austria).

ii  Toxicity Test of the Collagen-P(SSNa-co-GMAx) Cross-linked Network Hydrogels

The biocompatibility of prepared hydrogels was characterized as previously described [29,30]. In brief, small disks were produced from the cross-linked network hydrogels collagen-P(SSNa-co-GMAx), where x = 30 mol% GMA (Hydrogel 1) and x = 20 mol% GMA (Hydrogel 2), placed into 96-well plates, and then sterilized under UV irradiation. Then, nasal polyps fibroblasts of passage 7 in DMEM-10% FCS (2 × 104 cells per well) were seeded on hydrogels’ surface and cultured at 37 °C, under a 5% CO_2_ humidified atmosphere. Hydrogel disks were also placed in wells without cells and subjected to the same treatment as the corresponding wells containing gels and cells together, in order to be used as blank. After 24 h and 72 h of culture, the conditioned medium was removed and replaced by the same medium containing 1 mg/mL MTT (100 µL/well). The next steps were performed as described above. The net absorbance at 540 nm of wells, containing gels and cells together, was obtained after a subtraction of absorbance of wells containing only the gels.

### 2.6. Water-Uptake Measurements

In order to study the water-uptake capacity of the materials, 1 g of dry sample was soaked in 250 mL of deionized water for 6 days to be swelled completely. The weight of the hydrogel was measured after removing the excess of water (decantation). According to the weight of the swollen (*M*_2_) and dried (*M*_1_) samples, the water-uptake capacity (*Q*) was calculated according to the equation below:

(1) $$Q=\frac{M_{2}-M_{1}}{M_{1}}\times 100\%.$$

### 2.7. Release Degree of Oxidable Compounds in Water

The release degree of oxidable compounds (organic and inorganic) in distilled water of biofertilizers CH-Ref, CH-IN, and CH-Starch reflects their stepwise decomposition in aquatic medium in dynamic conditions [29]. A glass column of 2.8 cm inner diameter and 20 cm height was used, which was loaded with three layers of sand and gravel to ensure a uniform flow in the column among stagnant areas. Each layer had a height of 5 cm, arranged from the bottom to upwards, while the first gravel had the diameter between 6 and 9 mm, the second gravel between 4 and 6 mm, and the third between 0.4 and 1.8 mm. An amount of 5 g of each biofertilizer was placed on the sand layer. For each biofertilizer, two series of determinations was made. A constant flow of distilled water was ensured by connecting the column with a reservoir; the flow rate was 225 mL/h. Fractions of 100 mL were collected and the oxidable compound degree was determined using chemical oxygen demand CODMn method.

The initial weight of oxidable compounds was determined using a sample of 0.1 g of each fertilizer dissolved in 100 mL hot distilled water. The experiments occurred at 25 °C.

The quantitative evaluation of release degree (R) of oxidable compounds was performed by applying the relation:

(2) $$R=\frac{W_{i}}{W_{t}}\times 100$$

where R is the amount of oxidable compounds released (%), W_i_ is the amount of oxidable compounds released at t moment (g) and W_t_ is the total amount of oxidable compounds released (g).

## 3. Results

### 3.1. Synthesis and Characterization of P(SSNa-co-GMAx) Copolymers

**Figure 1:**
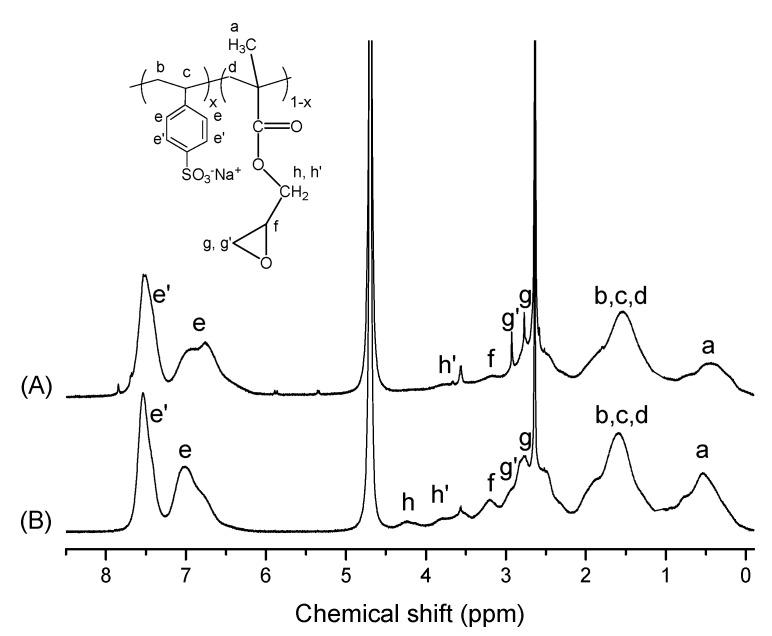
Proton Nuclear Magnetic Resonance (^1^H NMR) spectra of the copolymers poly(sodium 4-styrenesulfonate-co-glycidyl methacrylate) (P(SSNa-co-GMAx)) with 30 (**A**) and 40 (**B**) mol% GMA content in D_2_O.

The synthesis of the P(SSNa-co-GMAx) copolymers was conducted through free radical polymerization, as described previously, with x = 2, 5, 20, 30, and 40 mol% GMA. The chemical composition of the synthesized copolymers was estimated through ^1^H NMR spectroscopy. Figure 1 shows the ^1^H NMR spectra of the P(SSNa-co-GMAx) copolymers in D_2_O with 30 and 40 mol% GMA content. The peaks of GMA are clearly seen as shown by Figure 1A,B. The presence of SSNa from the aromatic (e, e’) protons at 6.0–7.8 ppm is clearly noticed at both spectra. Concerning GMA, the methylene protons of the epoxy ring of GMA correspond to the peak at 3.8 and 4.2 ppm (h’, h type protons), the methine proton was observed at 3.2 ppm (f type protons), and the two protons for the methylene of the ring were observed at 2.7 and 2.9 ppm (g, g’ type protons) [24,30]. Τhe protons (b, c, d) of the copolymer’s backbone are shown at 1.1–2.0 ppm and the methyl protons of GMA (a) at 0.5 ppm.

**Figure 2:**
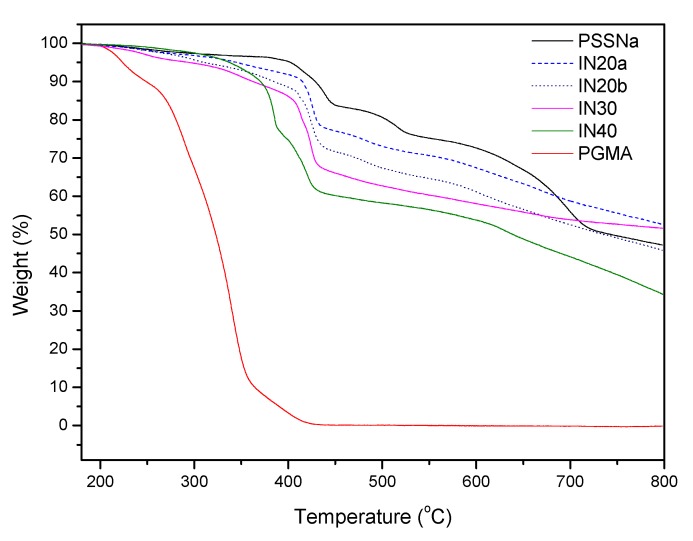
Thermogravimetric analysis (TGA) analysis of poly(sodium 4-styrenesulfonate) (PSSNa) and poly(glycidyl methacrylate) (PGMA) homopolymers and P(SSNa-co-GMA) copolymers with 20, 30, and 40 mol% GMA content.

Due to the fact that in some cases the peaks of GMA in the copolymers with GMA content lower than 30% are not clearly seen in ^1^H NMR spectra, thermogravimetric analysis was also applied in order to determine copolymers’ chemical composition. Figure 2 shows the thermal analysis of the copolymers IN20a, IN20b, IN30, and IN40, with 20, 30, and 40 mol% GMA content, respectively, as well as the homopolymers PSSNa and poly(glycidyl methacrylate) (PGMA). More specifically, since the PSSNa homopolymer is thermally stable up to 400 °C while PGMA shows thermal decomposition above 250 °C and its thermal decomposition is completed above 450 °C, the weight loss of the copolymers at 500 °C was considered to be related to GMA content. Based on the above consideration, GMA content was calculated taking into account the weight loss of the copolymers at 500 °C. The monomer feed composition and the characterization results from ^1^H NMR spectroscopy and TGA analysis of the P(SSNa-co-GMAx) synthesized copolymers are shown in Table 1.

**Figure 3:**
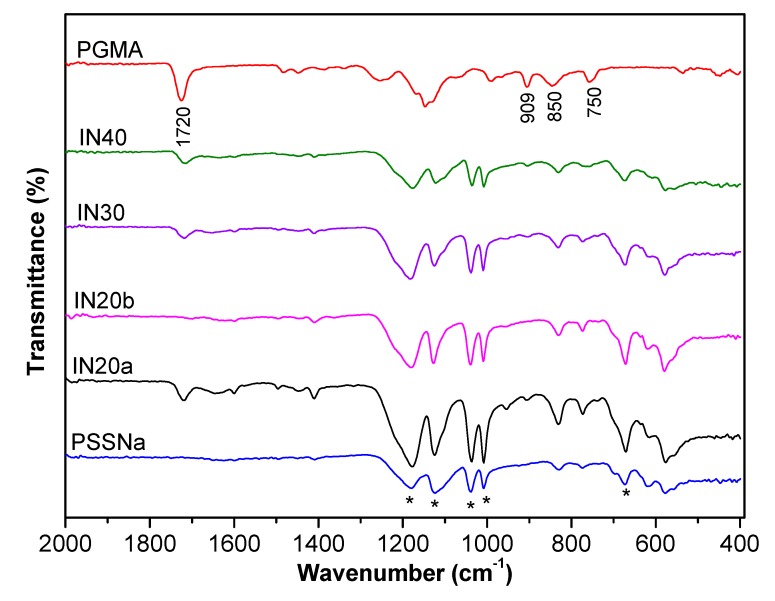
Attenuated Total Reflection Fourier Transform Infrared Spectroscopy (ATR-FTIR) spectra of P(SSNa-co-GMA) copolymers with 20 mol% (IN20a and IN20b), 30 mol% (IN30), and 40 mol% (IN40) GMA content. The spectra of the homopolymers PSSNa and PGMA are also reported.

The synthesized copolymers were examined in terms of chemical structure via ATR-FTIR spectroscopy and the spectra are presented in Figure 3. The spectra of poly(sodium 4-styrenesulfonate) (PSSNa) and poly(glycidyl methacrylate) (PGMA) homopolymers are also reported for comparison reasons.

The presence of PSSNa units in the copolymers was confirmed by the characteristic peaks shown with an asterisk. More specifically, the absorption band at 1010 and 1128 cm^−1^ assigned to the in-plane bending and the in-plane skeleton vibration of benzene ring, respectively. Moreover, the peaks at 1040 and 1182 cm^−1^ are related to the symmetric and antisymmetric vibration absorption peaks of SO_3_^−^ group, respectively. The peak at 676 cm^−1^ may be related to aromatic C–H out-of-plane bending (deformation) vibration [31,32]. The existence of the GMA unit in P(SSNa-co-GMAx) copolymers is verified by the characteristic band at 850 and 909 cm^−1^ assigned to the epoxy ring vibrations and the peak at 1255 cm^−1^ to the C−H of the epoxy group. The peak at 750 cm^−1^ corresponds to the C–H bending vibration of the epoxide ring. Moreover, in the region of 1720 cm^−1^ is the characteristic stretching of an ester group, related to the carbonyl group of the glycidyl methacrylate segment (C=O) [33,34]. As it may be seen from the ATR-FTIR spectra of Figure 3, the peak at 909 cm^−1^ of the epoxy group is obvious in all the copolymers.

**Figure 4:**
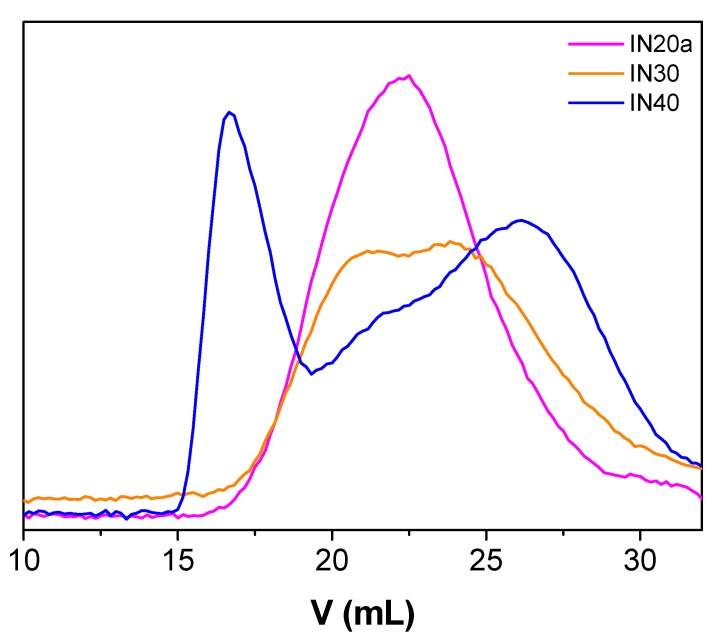
Size exclusion chromatography (SEC) curves for P(SSNa-co-GMAx) copolymers in water.

The molecular weight distributions of the P(SSNa-co-GMAx) copolymers were determined through size exclusion chromatography in aqueous 0.1 M LiNO_3_ solution, using poly(ethylene oxide) standards. The number-average molecular weights, Mn, weight-average molecular weights, Mw, and polydispersity indices, PDI, are presented in Table 2. Part of the results is also graphically shown in Figure 4. As we can see, high molecular weights (Mn values up to 83,000) were obtained and the polydispersity indices were quite high. In some cases, a bimodal distribution in the molecular weights and high DPn values were observed. The main driving force of this behavior is the Trommsdorff–Norrish effect, which induces an increase in the viscosity of the polymerizing medium leading to delayed reaction terminations. This in turn causes a rapid increase in the overall reaction rate, leading to a potential uncontrolled reaction and altering the characteristics of the produced polymer (auto acceleration) [35].

**Figure 5:**
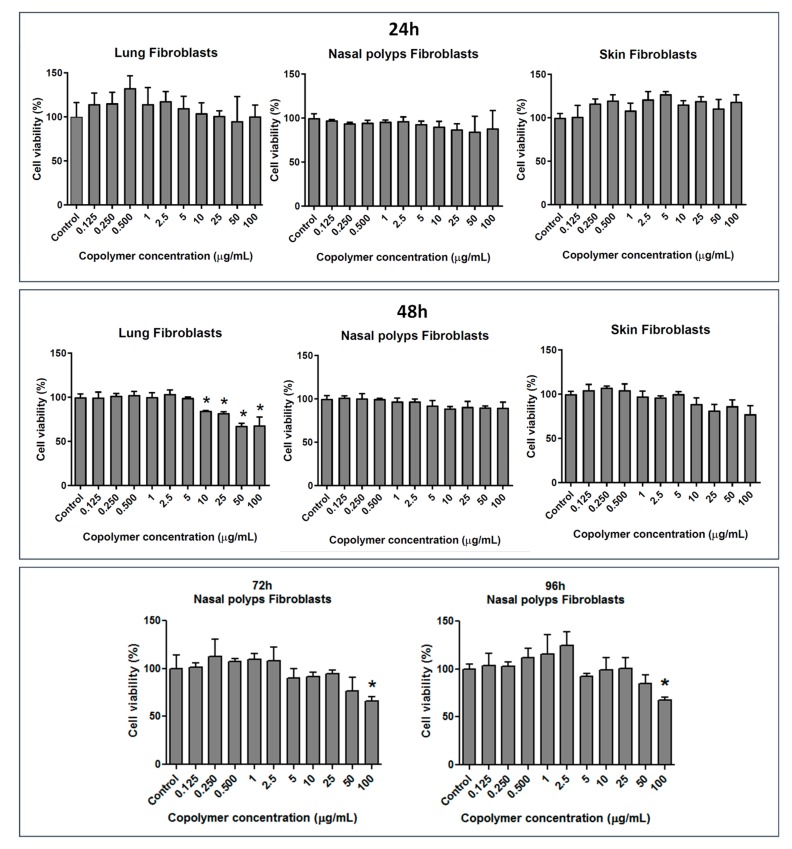
Effects of P(SSNa-co-GMA20) copolymer on lung, nasal polyps, and skin fibroblasts’ viability for different exposure periods. A range of concentrations from 0.125 to 100 μg/mL of copolymer was assayed. The results are expressed as mean ± SD of three separate experiments in triplicate. Statistically significant differences were evaluated using the unpaired Student’s t-test. (*) indicates statistically significant decrease (*p* < 0.05) compared to control.

The copolymer P(SSNa-co-GMA20) was then tested in terms of toxicity against normal lung and skin fibroblasts as well as against nasal polyps fibroblasts, and the cells viability was determined (Figure 5). These cells were selected because the corresponding organs and tissues are most likely to come into contact with the copolymer. As shown in Figure 5, the copolymer, independently of origination of cells, did not exhibit cytotoxic effects in all concentrations used at both exposure periods of 24 h and 48 h. Only at higher concentrations of copolymer (10–100 µg/mL) and for 48 h exposure period, a lower decrease of cells’ viability ranging from 15.3 to 31.8% for lung and 11.0 to 22.6% for skin fibroblasts, respectively, was observed, which, however, was significantly different compared to control in the case of lung fibroblasts (Figure 5, middle panel). The nasal polyps fibroblasts, the viability of which was not affected even at 100 µg/mL concentration of copolymer and 48 h exposure period, were further tested against the copolymer for longer exposure times 76 h and 92 h (Figure 5, lower panel). As shown in Figure 5 (lower panel), the copolymer, in both exposure times, caused statistically significant decrease of cells viability of about 30% only at the concentration of 100 µg/mL, supporting the suggestion that it does not exhibit cytotoxicity.

**Figure 6:**
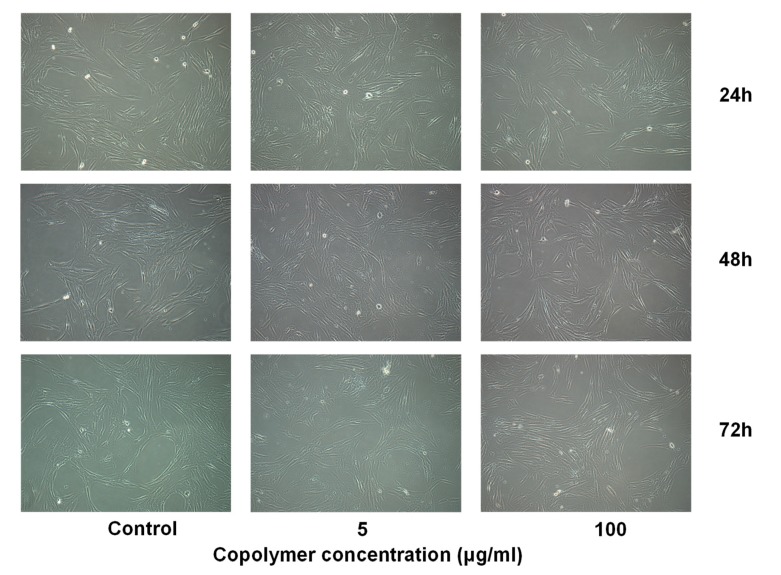
Phase contrast microscopy of nasal polyps fibroblasts cultured in the absence or presence of P(SSNa-co-GMA20) copolymer at concentrations 5 and 100 µg/mL for different exposure periods. All micrographs were taken at an original magnification of ×10.

In addition, the nasal polyps fibroblasts were cultured in the absence or presence of copolymer at concentrations 5 and 100 µg/mL for 24, 48, and 76 h, and the morphology of cells were examined by phase contrast microscopy on an Olympus CKX41 microscope carrying a MicroPublisher 3.3RTV camera (Figure 6). The microscopy revealed that the copolymer, at both concentrations used and in all exposure times, did not alter the cell morphology.

### 3.2. Preparation of the Collagen-P(SSNa-co-GMAx) Cross-Linked Network Hydrogels

**Scheme 1:**
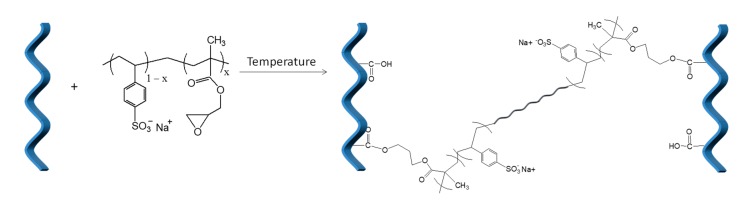
Cross-linking reaction of collagen hydrolysate and P(SSNa-co-GMAx) copolymers.

In order to develop materials that will efficiently incorporate the desired fertilizers by hydrogel formation, the collagen matrix was modified with functional polymeric electrolytes. Particularly, negatively charged polyelectrolytes were used as additives in order to interact with collagen hydrolysate, which in acidic conditions has a positively charged amino group and a carboxyl group. The cross-linking reaction of collagen hydrolysate and P(SSNa-co-GMAx) copolymers is schematically shown in Scheme 1. Based on our previous experience, we used the copolymer P(SSNa-co-GMAx), which has a dual functionality: the epoxy groups may create a chemical bond with the carboxyl groups of collagen hydrolysate and the SO_3_^−^ groups may electrostatically interact with the positively charged amino groups of collagen hydrolysate. For the formation of a cross-linking network between collagen hydrolysate and the P(SSNa-co-GMAx) copolymers, synthesized in the present work, various parameters were examined for the optimization of the reaction process.

Firstly, several experiments were conducted in order to test the difference between well-known water-soluble polymer electrolytes with the copolymers P(SSNa-co-GMAx), where x = 2, 5, 20, 30, and 40 mol% GMA. Thus, mixtures of the collagen hydrolysate with the homopolymers PSSNa, PAA, PEO and the copolymer poly(4-styrenesulfonic acid-co-maleic acid), and sodium salt P(SSNa-co-MA) were prepared in 80/20 (%) dry weight. These mixtures were stirred at R.T. for 1 h. Only in the case of PSSNa, a substantial quantity of a viscous mass was precipitated, separated from the supernatant solution and thermally treated at 80 °C for 20 h. The residue was determined around 60 wt%, which was eventually completely soluble in water.

The next step was to compare the polymer electrolyte PSSNa with the P(SSNa-co-GMAx) copolymers, where x = 2, 5, 20, 30, and 40 mol% GMA. Blends with collagen hydrolysate and the P(SSNa-co-GMAx) were prepared in the same conditions, as mentioned above. The thermal treatment was conducted at 80 °C for various time intervals. Thermal treatment at 120 °C was also tested for the reaction of collagen hydrolysate with the copolymer P(SSNa-co-GMA20).

Residues in the range of 70–100 wt% were obtained in all cases. Solubility tests where then applied to all residues and in the cases where swelling was observed and water-uptake measurements were performed. Representative results for the blends collagen hydrolysate/P(SSNa-co-GMAx) copolymers in 80/20 composition, after thermal treatment at 80 °C for 20 h, are shown in Table 3.

In the case of the copolymers with x = 2% and 5%, no swelling appeared since the immersion of the residue in excess water led to turbid solutions. On the other hand, P(SSNa-co-GMAx) with 20, 30, and 40 mol% GMA led to high residues insoluble in water. As it may be seen from Table 3, an higher water-uptake capacity was obtained when the copolymer P(SSNa-co-GMA20) was used. In the case of 30 and 40 mol% GMA, lower water-uptake capacities were observed showing that the GMA content in the copolymer may affect the swelling capacity of the cross-linked hydrogels.

Additionally, the quantity of the copolymer P(SSNa-co-GMA20) was investigated by varying the blend composition. In fact, blends of 99/1, 93/7, and 88/12 were compared with the blend 80/20 that was tested at first. From the results presented in Table 4, blend 99/1 led to a cloudy mass, whereas the rest compositions had a lower water retention compared to the composition 80/20. Experiments were also conducted with copolymers with 30 and 40 mol% GMA.

**Figure 7:**
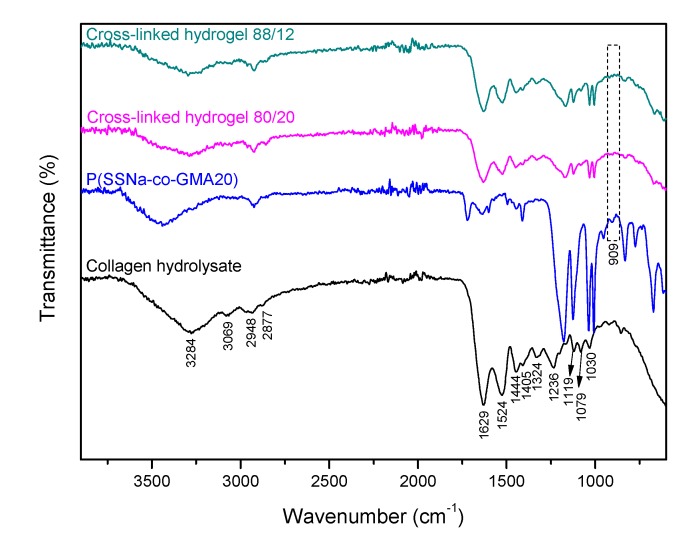
Attenuated Total Reflection Fourier Transform Infrared Spectroscopy (ATR-FTIR) spectra of dry collagen hydrolysate, copolymer P(SSNa-co-GMA20), and the cross-linked hydrogels obtained from the 80/20 and 88/12 blend compositions.

In Figure 7, the ATR-FTIR spectra of the dry collagen hydrolysate, the copolymer P(SSNa-co-GMA20), and the cross-linked hydrogel obtained from the 80/20 are presented. As it may be seen, the broad and intense peak at 3284 cm^−1^ is characteristic of the O–H and –NH_2_ groups of collagen hydrolysate. The existence of the transmittance band at 3069 cm^−1^ was related to the benzene ring. The bands appeared at 2948 and 2877 cm^−1^ are attributed to the –CH_2_ and –CH_3_ groups, respectively. In addition, the bands at 1629 and 1524 cm^−1^ correspond to the stretching vibration of C=O and bending vibration of N–H in the amide group (–CO–NH–), respectively [36]. The peaks at 1444 and 1405 cm^−1^ were assigned to the symmetric stretching vibrations of carboxylate (COO^−^) groups [4]. Moreover, the peak observed at 1324 cm^−1^ is due to the stretching vibration of –CH_2_ and bending vibration of C–H [37]. Another peak is located at 1236 cm^−1^ from the combination peaks between N–H deformation and C–N stretching vibrations [37,38]. Obviously, the bands obtained at 1119, 1079, and 1030 cm^−1^ represent stretching vibrations of C–O and C–O–C of ether groups. For the confirmation of the cross-linking reaction, we examined the peak of the epoxide group at 909 cm^−1^. As it may be seen from the spectrum of the cross-linked hydrogel, the peak was significantly decreased showing the successful reaction between collagen hydrolysate and epoxide groups of the copolymer P(SSNa-co-GMA20).

The curing temperature and time were also important parameters that needed to be investigated. For the cross-linking reaction between collagen and P(SSNa-co-GMA20) copolymers, the precipitated mass was left at the desired temperature (R.T., 80 °C or 120 °C) for a period of 15 min up to 20 h, depending on the curing temperature. According to a previous work that had been conducted by our group, curing at high temperatures would allow the epoxide ring opening of GMA [25]. When the curing of the precipitated mass was conducted at R.T., the cross-linked hydrogel was eventually completely soluble in water. Moreover, as mentioned above, the heat treatment was carried out for a period of 15 min up to 20 h, when the curing temperature was 80 °C, whereas at 120 °C, the tested curing period was 15 min to 2 h. The results for collagen hydrolysate/P(SSNa-co-GMA20) blends in composition 80/20 cured at 80 °C and 120 °C are presented in Table 5 and Table 6.

**Figure 8:**
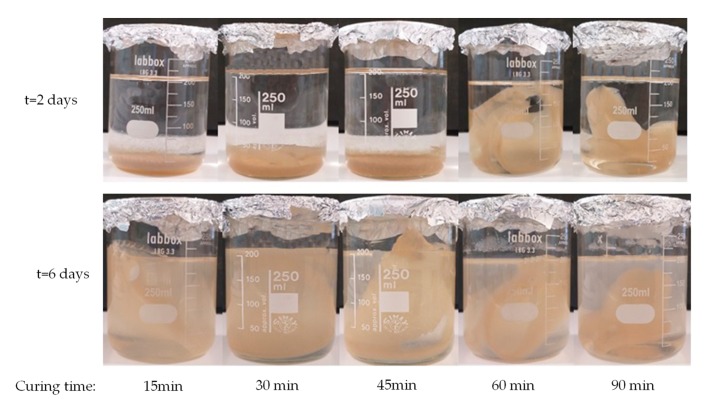
Visual observation of swelling of the cross-linked hydrogels cured at 120 °C (for 15–90 min) after 2 and 6 days.

**Figure 9:**
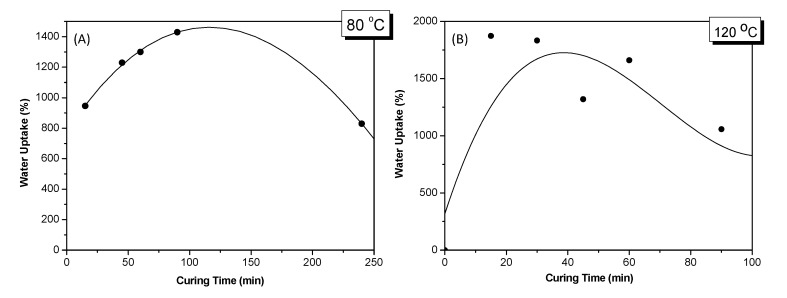
Water-uptake capacity of the cross-linked hydrogels after curing at (**A**) 80 °C and (**B**) 120 °C in various time intervals.

Finally, the water-uptake capacity of the cross-linked hydrogels was investigated. Examination of water absorbency behavior is essential for many applications in agriculture. As described in the experimental part, the hydrogels were immersed in excess water and left for 6 days. In Figure 8, a visual observation of the hydrogels cured at 120 °C at 2 and 6 days of the swelling period can be seen. As it may be seen, an increased swelling behavior was observed for all the hydrogels at the 6th day, even if a delayed swelling appeared for the ones cured at low time intervals (15 to 45 min). Almost the same attitude was observed for the hydrogels cured at 80 °C where in all cases the hydrogels showed high swellings after the 6th day (figure not shown). The water-uptake capacity of the cross-linked hydrogels cured at 80 and 120 °C at various time intervals are graphically presented at Figure 9A,B, where a third-order polynomial was applied for the curve fitting.

From the results of Table 5 and Figure 9A, we may notice the highest water-uptake capacity at curing time 90 min. Meanwhile, the results of Table 6 and Figure 9B showed a high value of water-uptake capacity at 15 min, which successively decreased with time. This behavior may be attributed to the fact that the cross-linking reaction accelerates at higher temperatures, and, thus in the case of 120 °C, higher water-uptake capacities were observed at lower curing times. The highest water-uptake capacity values observed in both curing temperatures indicate the formation of a cross-linked hydrogel network. The decrease of *Q* values after this point may be owed to a continuing cross-linking reaction leading to a more closed network that does not allow adequate swelling of the polymer hydrogels. Thus, the key point in the present work is not only to obtain high cross-linking ratios but also to achieve the ideal fraction between cross-linking and water-uptake capacity of the obtained hydrogels. In this point, it is very important to be able to control these factors according to the desired application.

Experiments conducted by Saruchi et al. [39] by using hydrogel interpenetrating polymer network (hydrogel-IPN) of natural gum polysaccharide with acrylamide and methacrylic acid showed swelling ratios between 165% and 259%. Moreover, Dang et al. [3] measured swelling rates for various blends of collagen protein powder with corn starch in water for 24 h reaching the maximum value of 89%. Li et al. [19] measured the swelling behavior of wheat straw cellulose hydrogels in various conditions, which showed values between 70 and 180 g/g.

Comparing the water-uptake capacities of the cross-linked hydrogels synthesized in the present work with the above-mentioned results published in literature, we may notice a very interesting behavior of the current materials since they show higher water-uptake values in the range of 800–1800% at short time intervals. The results are very encouraging in order to proceed to a further investigation of these or other systems.

**Figure 10:**
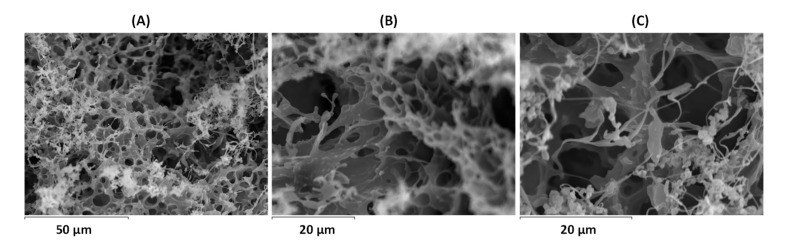
Scanning Electron Microscopy (SEM) images of cross-linked hydrogels for blends of collagen hydrolysate/P(SSNa-co-GMA30) and after thermal treatment at 80 °C for magnifications of 1000× (**A**), 2000× (**B**), and 2700× (**C**).

The morphological characterization of the cross-linked hydrogels for the blends of collagen hydrolysate/P(SSNa-co-GMA30) was conducted through Scanning Electron Microscopy (SEM). After the thermal treatment at 80 °C and swelling for 6 days, the hydrogels were obtained through freeze-drying for SEM characterization. Figure 10 shows the micrographs of the cross-linked hydrogels. From magnifications of 1000× (Figure 10A), 2000× (Figure 10B), and 2700× (Figure 10C). The surface of the samples revealed a 3-D structure with a porous morphology with pore size ranging from 1–6 μm.

**Figure 11:**
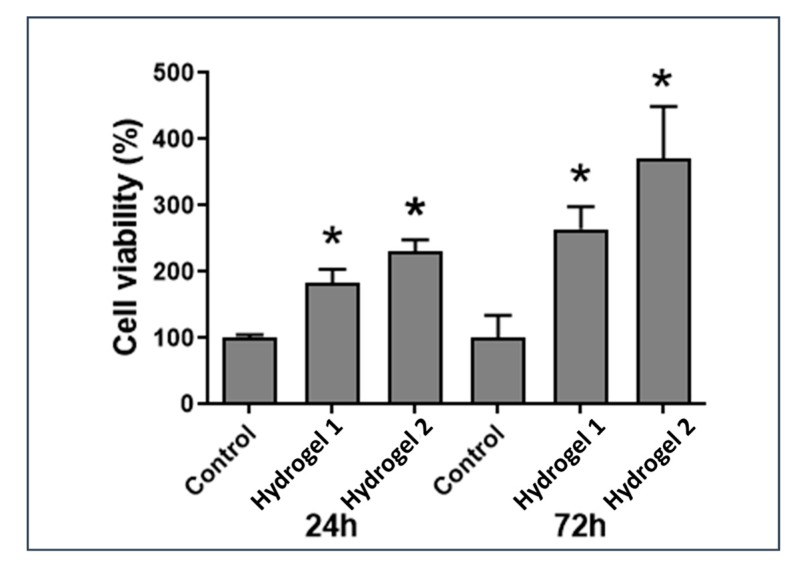
Evaluation of cell viability after 24 and 72 h culture of cells in contact with hydrogels. Control: cells in the absence of hydrogels. Each result is the mean ± SD of at least three independent experiments. Statistically significant differences were evaluated using the unpaired Student’s t-test. (*) indicates statistically significant increase (*p* < 0.05) compared to control.

The hydrogel’s cytotoxicity was also tested against the nasal polyps fibroblasts (Figure 11). As shown in Figure 11, the fibroblasts viability was not affected after 72 h of being in contact with hydrogels. On contrary, in the presence of materials, a significant increase of cells proliferation was observed. It should also be noticed that the degradation by-products that could be produced during the 72 h incubation did not affect cell viability.

### 3.3. Characterization of Collagen Hydrogel Fertilizers

Chemical modification of collagenous materials may occur with a micro- or macromolecular reagent, capable of interacting with one or more types of functional groups attached to the polypeptide chain. The encapsulation of nutrients into the collagenous materials and the modification with functional polymers led to the development of hydrogels that may be applied as potential fertilizers. Based on this methodology, the copolymer P(SSNa-co-GMA20) was used in order to ensure functionality with the collagenous materials containing nutrients. In parallel, starch was used as a biopolymer in combination with the cross-linker methylene bis-acrylamide for the modification of the nutrient encapsulated collagen. Various parameters, such as temperature and polymer concentration, were examined in order to obtain the optimum conditions for the functionalization of enriched collagen hydrogels with functional polymers in order to obtain biofertilizers with controlled release.

Chemical analysis of the three fertilizers (Table 7) reveals similar features between reference sample and the sample functionalized with the synthetic copolymer IN20c, while the collagen hydrolysate functionalized with starch shows minimum content in dry substance, ash, and total nitrogen.

**Figure 12:**
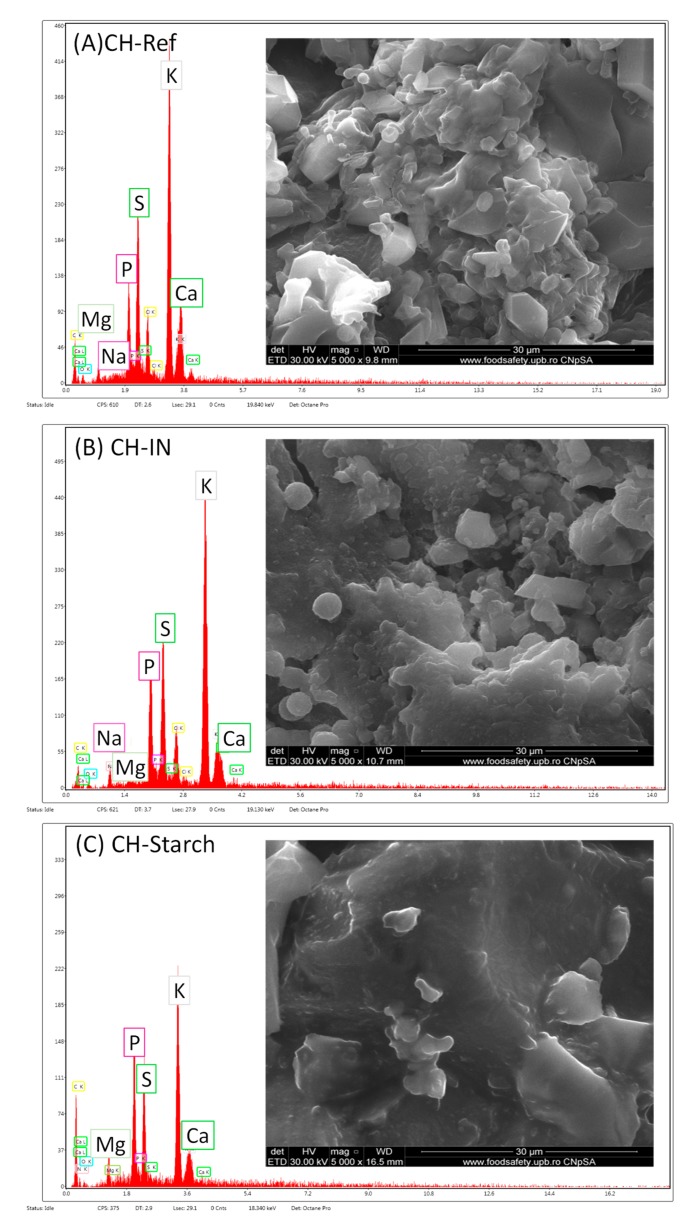
Energy Dispersive X-ray analysis-Scanning Electron Microscope (EDAX-SEM) analysis and microstructure of (**A**) CH-Ref, (**B**) CH-IN, and (**C**) CH-Starch.

The fertilizing capacity is conferred mainly by the content of the nutrients encapsulated, quantified in Table 8. Their presence is confirmed by EDAX analysis (Figure 12A–C), where the signal specific to potassium is dominant in all three samples, while the presence of other useful elements is detected, such as phosphorous, sulfur, magnesium, calcium, sodium, etc. A different note among the samples is manifested by the carbon content, which is the highest for collagen hydrolysate functionalized with starch, as expected. Moreover, examination of SEM images reveals similar microstructures for CH-Ref and CH-IN samples (Figure 12A,B) that could be explained by quite a low content of synthetic copolymer (5% IN20c) imposed by biodegradability requirements of fertilization, which does not create major changes in the collagen microstructure. At the same time, the high content of highly biodegradable biopolymer starch results in important changes in the morphology of these samples as compared with the reference sample (Figure 12C).

**Figure 13:**
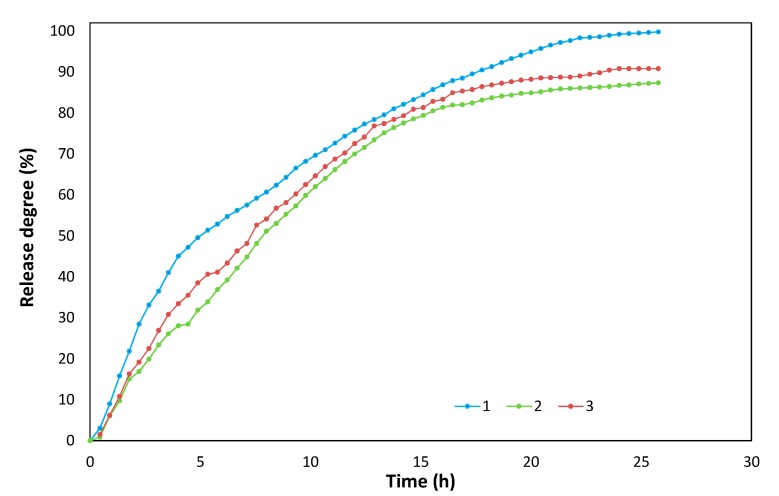
Evolution in time-of-release degree of oxidable compounds in free water for biofertilizers CH-Ref (1), CH-IN (2), and CH-Starch (3).

In Figure 13, the variation of release degree for oxidable compounds in time for these biofertilizers is illustrated.

Initial content of oxidable compounds, CODMn, was 287.4, 336, and 382 mg O_2_/g for CH-Ref, CH-IN, and CH-Starch biofertilizers, respectively.

The experimental data concerning the release degree of oxidable compounds in free water of these biofertilizers increases in time to maximum after about 26 h: 99.9%, 87%, and over 90%, for CH-Ref, CH-IN, and CH-Starch, respectively (Figure 13). Reference collagen hydrolysate exhibits a higher capacity to release of oxidable compounds as compared to the other two samples, thus revealing that functionalization of collagen results in slowing their release capacity.

As referring to the release mechanism, one may suppose that in the first stage, the organic and inorganic compounds with high solubility are removed, like peptides with short chains, soluble polymers (starch), aminoacids, glucides (mono- and di-zaharides), nitrate salts, acids, etc. In the second stage, the rate of release of oxidable compounds is decreasing for all fertilizers and can be assessed to organic compounds with low solubility, such as proteins, collagen, and degradable polymers [29].

## 4. Conclusions

The combination of collagen hydrolysate as a matrix with the water-soluble polymers provides the development of cross-linked network hydrogels with the prospect to obtain “smart” organic biofertilizers. In the present work, copolymers with various GMA content were synthesized and their characterization in terms of stoichiometry, thermal behavior, molecular weight, and chemical structure was performed. It was also assessed that the prepared copolymer, as well as the respective hydrogels, was not cytotoxic against fibroblasts of different origin, normal or pathologic, and it did not alter the cells’ morphology. In a further step, various blends of collagen hydrolysate with different functionalized water-soluble polymers were made in order to prepare cross-linked network hydrogels. ATR-FTIR analysis confirmed the cross-linking reaction between collagen hydrolysate and epoxide groups of the copolymer. Depending on the cross-linking ratio, we were able to control the water-uptake capacity of the cross-linked hydrogels.

Moreover, preliminary tests on three samples of collagen hydrolysate with encapsulated nutrients (N, P, K), functionalized with the synthetic copolymer and starch as biopolymer, led to the development of hydrogels that may be applied as potential fertilizers. The variation of release degree for oxidable compounds in time for these biofertilizers showed that the enriched collagen functionalized with the polymers led to a slower release capacity of oxidable compounds than the un-functionalized enriched collagen.

It is worth to note that the current work presents a concept with high environmental impact, since the recovery of collagen hydrolysate from tannery waste contributes to a circular economy model involving socio-economic advantages. Moreover, the P(SSNa-co-GMAx) is a cost-effective copolymer since the monomers may be provided in bulk quantities with low cost.

These findings provide new insights and foster a better understanding of the behavior of the cross-linked network hydrogels based on the combination of collagen hydrolysate and hydrophilic polymers with functional groups, which may open the way for new practical applications of these systems in agriculture as a controlled-release biostimulant for plant growth.

## Figures and Tables

**Table 1:** Characterization results for the P(SSNa-co-GMAx) copolymers.

Copolymers	Code	Feed Composition (mol% GMA)	^1^H NMR Composition (mol% GMA)	TGA Composition (mol% GMA)
P(SSΝa-co-GMA2)	IN2	2	2	2
P(SSΝa-co-GMA5)	IN5	5	6	6
P(SSΝa-co-GMA20)	IN20a	20	20	20
P(SSΝa-co-GMA20)	IN20b	20	15	20
P(SSΝa-co-GMA20)	IN20c	20	13	20
P(SSΝa-co-GMA20)	IN20d	20	15	19
P(SSΝa-co-GMA20)	IN20e	20	12	19
P(SSΝa-co-GMA30)	IN30	30	29	30
P(SSΝa-co-GMA40)	IN40	40	42	40

**Table 2:** Size exclusion chromatography (SEC) characterization for P(SSNa-co-GMAx) copolymers.

Sample	P(SSNa-co-GMAx)	Solubility in Water	M_n_	M_w_	DP_n_
IN20a	20	+++	83,000	360,000	4.3
IN20b	20	+++	71,000	398,000	5.6
IN20c	20	+++	69,900	1,100,000	15.6
IN20d	20	+++	63,000	371,000	5.9
IN30	30	++	41,500	147,000	3.5
IN40	40	++	30,000	372,000	12.2

+++: Excellent, ++: Very good, +: Good.

**Table 3:** Residues and water-uptake capacities for blends of collagen hydrolysate/P(SSNa-co-GMAx) copolymers in 80/20 composition after thermal treatment at 80 °C for 20 h.

GMA (mol% A)	Residue, R (%)	Water-Uptake Capacity, Q (%)
2	77	Cloudy mass
5	70	Cloudy mass
20	87	476
30	100	416
40	71	438

**Table 4:** Water-uptake capacities for blends of collagen hydrolysate/P(SSNa-co-GMA20) in various compositions and thermal treatment at 80 °C for 20 h.

Collagen Hydrolysate (wt%)	P(SSNa-co-GMA20) (wt%)	Water-Uptake Capacity, Q (%)
99	1	Cloudy mass
93	7	157%
88	12	213%
80	20	476%

**Table 5:** Residues and water-uptake capacities for blends of collagen hydrolysate/P(SSNa-co-GMA20) in composition 80/20 at various time intervals and curing temperature 80 °C.

Thermal Treatment of the Residue	Residue (%)	Water-Uptake Capacity, Q (%)
15 min	82	946
30 min	79	833
45 min	82	1230
60 min	79	1300
90 min	81	1430
120 min	82	696
4 h	81	830
20h	82	476

**Table 6:** Residues and water-uptake capacities for blends of collagen hydrolysate/P(SSNa-co-GMA20) in composition 80/20 at various time intervals and curing temperature 120 °C.

Thermal Treatment of the Residue	Residue (%)	Water-Uptake Capacity, Q (%)
15 min	81	1874
30 min	81	1834
45 min	82	1312
60 min	83	1661
90 min	82	1058
120 min	81	996

**Table 7:** Chemical analysis of fertilizers.

Characteristics	Sample Code			Standard Used
	CH-Ref	CH-IN	CH-Starch	
Dry Substance, %	61.22	65.80	21.59	SR EN ISO 4684: 2006
Ash, %	22.36	25.18	17.23	SR EN ISO 4047: 2002
Total nitrogen, %	10.55	10.14	8.29	SR ISO 5397: 1996
pH	7.20	6.87	6.76	STAS 8619/3: 1990

**Table 8:** Nutrient content of fertilizers.

Characteristics	Sample Code			Standard Used
	CH-Ref	CH-IN	CH-Starch	
Total N, %	10.55	10.14	8.29	SR ISO 5397: 1996
P_2_O_5_, %	7.67	6.75	5.54	SR EN 15959/2012
K_2_O, %	10.07	8.21	10.05	SR ISO 5397: 1996
